# Personal data governance and privacy in digital reproductive, maternal, newborn, and child health initiatives in Palestine and Jordan: a mapping exercise

**DOI:** 10.3389/fdgth.2023.1165692

**Published:** 2023-05-25

**Authors:** Maysaa Nemer, Yousef S. Khader, Mohammad S. Alyahya, Alexandrine Pirlot de Corbion, Sundeep Sahay, Niveen M. E. Abu-Rmeileh

**Affiliations:** ^1^ Institute of Community and Public Health, Birzeit University, Birzeit, Palestine; ^2^ Department of Community Medicine, Public Health and Family Medicine, Faculty of Medicine, Jordan University of Science & Technology, Irbid, Jordan; ^3^ Department of Health Management and Policy, Faculty of Medicine, Jordan University of Science and Technology, Irbid, Jordan; ^4^ Privacy International, London, United Kingdom; ^5^ Department of Informatics, University of Oslo, Oslo, Norway; ^6^ HISP India, New Delhi, India

**Keywords:** digital health initiatives, e-health, m-health, e-registry, health information systems, RMNCH, Palestine, Jordan

## Abstract

**Introduction:**

There is a rapid increase in using digital technology for strengthening delivery of reproductive, maternal, newborn, and child health (RMNCH) services. Although digital health has potentially many benefits, utilizing it without taking into consideration the possible risks related to the security and privacy of patients' data, and consequently their rights, would yield negative consequences for potential beneficiaries. Mitigating these risks requires effective governance, especially in humanitarian and low-resourced settings. The issue of governing digital personal data in RMNCH services has to date been inadequately considered in the context of low-and-middle-income countries (LMICs). This paper aimed to understand the ecosystem of digital technology for RMNCH services in Palestine and Jordan, the levels of maturity of them, and the implementation challenges experienced, particularly concerning data governance and human rights.

**Methods:**

A mapping exercise was conducted to identify digital RMNCH initiatives in Palestine and Jordan and mapping relevant information from identified initiatives. Information was collected from several resources, including relevant available documents and personal communications with stakeholders.

**Results:**

A total of 11 digital health initiatives in Palestine and 9 in Jordan were identified, including: 6 health information systems, 4 registries, 4 health surveillance systems, 3 websites, and 3 mobile-based applications. Most of these initiatives were fully developed and implemented. The initiatives collect patients' personal data, which are managed and controlled by the main owner of the initiative. Privacy policy was not available for many of the initiatives.

**Discussion:**

Digital health is becoming a part of the health system in Palestine and Jordan, and there is an increasing use of digital technology in the field of RMNCH services in both countries, particularly expanding in recent years. This increase, however, is not accompanied by clear regulatory policies especially when it comes to privacy and security of personal data, and how this data is governed. Digital RMNCH initiatives have the potential to promote effective and equitable access to services, but stronger regulatory mechanisms are required to ensure the effective realization of this potential in practice.

## 1. Introduction

The use of digital technology in the healthcare sector has widely accelerated all over the world (1), with diverse digital interventions being introduced in reproductive, maternal, newborn, and child health (RMNCH) services including registries, health surveillance systems, telehealth, and mobile applications (m-health) (1–5). In some cases, these interventions have played a role in improving the utilization and access to health services and in strengthening coordination between different service providers (6–10). It has been argued that these initiatives have helped expand the coverage of health services and overcome the geographical, social, and behavioral barriers, exemplifying their positive potential for enhancing the RMNCH services delivery (11).

In recent years, the use of digital health in RMNCH has particularly increased in low-and-middle-income countries (LMICs) (8, 10, 12). The transition from paper-based to electronic-based registries and the availability of free and open-source tools and frameworks have the potential to facilitate progress in the management of health information (7). Several studies have evaluated the effectiveness of the use of these digital tools for the delivery of RMNCH services, including the use of mobile applications for family planning (9) and for antenatal care services (13). However, a limited evidence of their impacts on health outcomes among patients, emphasizing the need for further research, has been discovered (8).

Further, a limited research on digital health for RMNCH services in Palestine and Jordan, particularly related to issues of data governance, human rights, and gender equality, has been established. One study has described the implementation of an e-registry for obstetric interventions and childbirth events in the West Bank and Gaza (14). In addition, a randomized control trial in Palestine demonstrated the effect of a contraceptive behavioral intervention, in which women received mobile text messages with instructions on possible contraceptive methods, and examined women's attitudes toward effective contraception (15). Other studies include the introduction of the electronic health records (e-health system) of the United Nations Relief and Works Agency for Palestine Refugees in the Near East (UNRWA) to support their different health services such as maternal and child health (16). Moreover, the UNRWA has released the electronic “Maternal and Child Health Handbook” application in Jordan, to give pregnant women and mothers access to educational information and health records on smartphones (17).

Although digital health has potentially many benefits, utilizing it without taking into consideration the possible risks related to the security and privacy of patients’ data, and consequently their rights, would yield negative consequences for potential beneficiaries. Mitigating these risks requires effective governance, especially in humanitarian and low-resourced settings (11, 18–22). To date, the issue of governing digital personal data in RMNCH services has been inadequately considered in the context of LMICs.

This paper seeks to address this important research gap by seeking to understand the ecosystem of digital technology for RMNCH services in Palestine and Jordan, the levels of maturity related to implementation and evaluation, and the main challenges experienced, particularly in relation to issues of data governance and human rights.

## 2. Methods

### 2.1. Study design

A mapping exercise was conducted to identify digital RMNCH initiatives in Palestine and Jordan by using a structured tool.

### 2.2. Search strategy

After an initial exploration which identified the absence of centralized platform and/or public, private, and non-state stakeholders or authority responsible for digital RMNCH initiatives, the research team deployed various techniques over a 3-month period (March–May 2020) to identify such initiatives ongoing in Palestine and Jordan.

First, a general open-source search was performed using search engines such as Google, Bing, and Yahoo to identify relevant initiatives. Second, Google Scholar, PubMed, and Medline were searched for relevant studies or using data based on these initiatives. A wide range of keywords was used to guide the search such as: ([“women” or “children” or “mother” or “reproductive health” or “maternal health” or “newborn health” or “child health”] and [“health information system” or “ehealth or “mhealth or “mobile health” or “digital health” or “registry” or “electronic surveillance”] and [Jordan (Country) or Palestine]). Third, key stakeholders such as providers of RMNCH services especially gynecologists, pediatricians, lab technicians, and insurance and IT companies associated with RMNCH services were identified. The websites and social media feed channels of these organizations were searched (using Arabic and English keywords) to obtain more detailed information (such as annual reports) on their digital health activities using both English and Arabic search terms. Terms used were electronic health, digital health, and health systems. For Arabic websites, words like “hawkamah” or “hawsaba” were used as search terms. Fourth, for private sector entities such as clinics, hospitals, laboratories, and insurance companies, a paragraph in Arabic was posted on many IT Facebook pages, such as the Palestine's Information and Communications Technology Incubator (PICTI) and PalGeeks. In the post, they were asked to contact the research team if they were involved in or knew of any organization that has developed digital health initiatives in the country so that they would be contacted to get more information. Fifth, several related individuals were contacted, such as health professionals, academics, IT specialists, and researchers, and were probed on their awareness of such digital initiatives.

### 2.3. Inclusion criteria

Only those initiatives which concerned digitization efforts around RMNCH services, for both individual and aggregate data, were included for the analysis. This included health information systems (HIS), registries, health surveillance systems, mobile applications, and websites. Initially, all initiatives identified were included, and then based on discussions and consensus between the research team members, the final list of initiatives were selected for detailed analysis.

### 2.4. Mapping tool

A mapping process was performed by two researchers from each country and lasted for 3 months (June–August 2020). The research team developed an extraction sheet for mapping relevant information from the selected digital RMNCH initiatives which included the following variables: (i) name of the initiative, (ii) data source, (iii) the implementing agency, (iv) the funding or supporting agency, (v) objectives of the initiative, (vi) time and place of implementation, (vii) whether in primary healthcare or hospital settings or both, and (viii) target population and beneficiaries.

The data were retrieved from different sources, such websites, reports, news, and events, from social media platforms of institutions, research articles, and available policy documents. The data that could not be found online were collected using personal communications via phone calls or by meeting relevant stakeholders, such as managers and IT staff.

## 3. Results

### 3.1. Digital RMNCH initiatives in Palestine and Jordan

A total of 26 digital health initiatives in Palestine and 15 in Jordan were identified. Of those, 20 (11 in Palestine and 9 in Jordan) met the inclusion criteria and were included in the analysis. The breakdown of the initiatives included was as follows: (i) six HISs, (ii) four registries, (iii) four health surveillance systems, (iv) three websites, and (v) three mobile-based applications. Table 1 details these different initiatives, while Table 2 summarizes their key characteristics.

**Table 1:** Types of the main digital reproductive, maternal, newborn, and child health (RMNCH) initiatives in Palestine and Jordan.

Type	Palestine	Jordan
Health information system (HIS)/electronic health record (EHR)	1. Avicenna system 2. Family Health Team Approach and the e-health system reform–United Nations Relief and Works Agency (UNRWA)	3. Hakeem 4. Family Health Team Approach and the e-health system reform–United Nations Relief and Works Agency (UNRWA) and e-MCH handbook 5. Health information systems at King Abdullah University Hospital (I-Soft) 6. Health information system in private hospitals CertaCure
Registry	7. Maternal and child health e-registry (MCH e-registry) 8. Mammogram e-registry 9. NutriDash (UNICEF)	10. Harmonized reproductive health registry (*h*RHR)
Health surveillance system	11. Maternal Mortality Surveillance 12. National Observatory for Gender-Based Violence (GBVO)	13. Jordan's Maternal Mortality Surveillance and Response System (JMMSR) 14. Jordan Stillbirth and Neonatal Death Surveillance System (JSANDS)
Website/YouTube (online)	15. Electronic page and YouTube channel for educational and promotional material for maternal and child health (MCH) 16. WebTeb website	17. The Breastfeeding Support Association Website
Mobile application/short message service (SMS)	18. Nabd Al-Hayat application 19. Sawa 121 “Child Protection Helpline 121”	20. Children Immunization Application (CImA)

**Table 2:** Description of the digital reproductive, maternal, newborn, and child health (RMNCH) initiatives available in Palestine and Jordan.

No.	Initiative	Country	Implementer	Owner	Funder	Year of development	Sector (public, private, UN, NGOs)	Main objective	Status (active or not)	Had been evaluated (yes/no)	Program or research project
1	Avicenna system	West Bank only	Ministry of Health (MOH)	MOH	MOH, United States Agency for International Development (USAID)	2010–2011	Public hospitals	To provide a common source of information about a patient's health history to enhance the ability of healthcare professionals to coordinate care by providing a patient's health information and visit history at the place and time that it is needed	Active	Yes, the systems’ data completeness and reliability and adoption by users were evaluated	Program
2	Family Health Team Approach and the e-health system reform– UNRWA	West Bank, Gaza, and Jordan	United Nations Relief and Works Agency (UNRWA)	UNRWA	United States, Denmark, Switzerland, Japan, the Saudi Fund for Development, and Arab–Brazilian Chamber of Commerce (CCAB), based in São Paulo, Brazil	2013	UN	Reduce staff workload, improve daily operations (data recording and reporting), improve the quality of data, reduce medical errors, reduce paperwork, and improve clinical staff capacities and performance	Active	Yes, the quality of the information inserted into or generated from the e-health system, the quality of the e-health system, the perceived usefulness and ease of use, and system performance were evaluated	Program
3	Hakeem	Jordan	Electronic Health Solutions (EHS)	MOH, Ministry of Digital Economy and Entrepreneurship (MODEE), Royal Medical Services (RMS), King Hussein Cancer Foundation (KHCF), Royal Health Awareness Society (RHAC), Private Hospitals Association (PHA), and King Abdulla II Fund for Development (KAFD)	EHS	2009	Public hospitals and centers	Provide a common source of information about a patient's health history to enhance the ability of healthcare professionals to coordinate care by providing a patient's health information and visit history at the place and time that it is needed	Active	Yes, the effectiveness of the health system according to the international health information systems standards, some internal factors of the system, the data collection mechanisms, and the attitudes of healthcare professionals toward adopting this HIS were evaluated	Program
4	Electronic Mother and Child Health (e-MCH) handbook	Jordan	UNRWA	Japan International Cooperation Agency (JICA)	UNRWA and JICA	2017	UN	e-MCH is a smartphone software application of the MCH handbook to increase both the continuum of care and access to maternal and child health services, send its user reminders of appointment times, provide health education information, and function as a more efficient communication tool between patients and UNRWA health centers	Active	Yes. the dissemination and utilization status of e-MCH by Palestine refugees in Jordan was evaluated by a cross-sectional evaluation study 3 months after its introduction	Program
5	Health Information System at King Abdullah University Hospital (I-Soft)	Jordan	King Abdullah University Hospital (KAUH)	KAUH	KAUH	2004	Public hospitals	An enterprise class, fully integrated EHR software solution for providers to support acute, ambulatory and emergency care settings	Active	No	Program
6	CertaCure	Jordan	CertaCure	CertaCure	CertaCure	2016	Private hospitals	CertaCure is a comprehensive, integrated hospital information system (HIS) designed to manage hospitals’ operations, such as medical, administrative, and all aspects	Active	No	Program
7	Maternal and child health e-registry (MCH e-registry)	West Bank and Gaza	Palestinian National Institute of Public Health (PNIPH), World Health Organization (WHO), MOH	MOH	Norwegian representative in Palestine, the Norwegian Institute of Public Health (NIPH), Palestinian National Institute of Public Health (PNIPH)	2014	Public centers	To transfer maternal and child personal data from the clinical level to the national level to support evidence-based decision-making, including antenatal, postnatal, and newborn health services	Active	Yes, the roles of this registry in improving quality of care and health of pregnant women and their babies, the role of it in improving time-efficiency by care providers, and if the routine health information systems indicators can change with the introduction of the e-registry were evaluated	Program
8	Mammogram e-registry	West Bank and Gaza	Palestinian National Institute of Public Health (PNIPH) and close collaboration with the Ministry of Health (MOH)	Ministry of Health (MOH)	NIPH, University of Oslo (UiO), PNIPH	2017–2018	Public hospitals and centers	To monitor and track information about all women undergoing mammogram screening	Active	No	Program
9	NutriDash (UNICEF)	Global initiative, includes West Bank and Gaza	United Nations International Children's Emergency Fund (UNICEF), in partnership with local organizations	UNICEF	Mainly funded by UNICEF and other donors	2013	UN	Track data for nutrition programs and compare and analyze nutritional development initiatives around the world to understand priorities and nutritional needs for populations. At the program level, UNICEF NutriDash platform enables countries to report on the performance of nutrition programs worldwide	Active	No	Program
10	Harmonized reproductive health registry (*h*RHR)	Jordan	MOH	The International Development Research Centre (IDRC)	EMPHNET	2019	Public hospitals and centers	To improve access to and use of reproductive health data in order to facilitate program planning and policy development	Pilot	Ongoing	A pilot research project
11	Maternal Mortality Surveillance	West Bank	MOH	MOH	MOH	2010	Public hospitals	Register maternal deaths and their causes	Active	No	Program
12	National Observatory for Gender-Based Violence (GBVO)	West Bank and Gaza	Ministry of Women’s Affairs (MWA)	MWA	Italian Agency for Development Cooperation, PNIPH, and MOH	2018–2020	Public hospitals and centers	To monitor and analyze gender-based violence or violence against women in all its forms	No	No	Program
13	Jordan's Maternal Mortality Surveillance and Response System (JMMSR)	Jordan	MOH	USAID	MOH and USAID	2018	Public	Register maternal deaths and causes	Active	Yes, the system's functions, data accuracy, and timeliness of notifying, identifying, and reviewing all maternal death cases were evaluated	Program
14	Jordan’s Stillbirth and Neonatal Death Surveillance System (JSANDS)	Jordan	MOH	IDRC and UNICEF	JUST and MOH	2019	Public	An online data entry system to collect, organize, analyze, and disseminate data on stillbirths, neonatal deaths, and their causes	Active	Yes (ongoing), the system's usefulness, functionality, and other attributes were evaluated	A pilot research project
15	Electronic page and YouTube channel for educational and promotional material for MCH	West Bank and Gaza	MOH, UNRWA, and Ministry of Education (MOE)	MOH	MOH, WHO, UNFPA, World Vision, Italian Cooperation	2016	Public	To increase community health awareness especially about MCH	Active	No	Program
16	WebTeb Website	Palestine, Jordan, Dubai, online	Web Medicine Limited Company	Web Medicine Limited Company		2011	Private	Make the trusted health and medical information available for Arabic reader everywhere and aimed to be an Arabic platform connecting between the Arabic citizens and different health information providers worldwide	Active	No	Program
17	The Breastfeeding Support Association Website	Jordan	The Breastfeeding Support Association	The Breastfeeding Support Association	The Breastfeeding Support Association	2016	Public	To disseminate related information about breastfeeding and communicate with mothers to ask inquiries or share their experiences	Active	No	Program (website)
18	Nabd Al- Hayat application	West Bank and Gaza, online	Arab American University in Palestine (AAUP) and MOH	AAUP, MOH	AAUP, World Vision	2018	Public	Increase health awareness regarding maternal and child health (MCH)	Pilot testing	No	Program
19	Sawa 121 “Child Protection Helpline 121”	West Bank, Jerusalem, 1948 lands, Gaza Strip	Sawa “All women together today and tomorrow”	Sawa “All women together today and tomorrow”	UNICEF, UNFPA, and other European funders	2004	Private	A hotline with database application embedded to collect information on cases who are asking for help in counseling/guidance/support, especially children and women, suffering from violence, abuse, or other forms of mistreatment and distress	Active	Yes, performance was evaluated	Program
20	Children Immunization Application (CImA)	Jordan, Syrian refugees in Zaatari camp	MOH	Grand Challenges Canada and the Karolinska Institutet Foundations and Funds	Jordan University of Science and Technology (JUST) and Karolinska Institutet, Stockholm, Sweden	2019	Public	Support parents in fully documenting the vaccine history of their children and to trace defaulters	Active	Yes, the impact of the app was evaluated	A pilot research project

MOH, Ministry of Health; PNIPH, Palestinian National Institute of Public Health; MWA, Ministry of Women's Affairs; WHO, World Health Organization; UNRWA, United Nations Relief and Works Agency; UNICEF, United Nations International Children's Emergency Fund; UNFPA, United Nations Population Fund; UN, United Nations; UiO, University of Oslo; NIPH, Norwegian Institute of Public Health; GBV, Gender-Based Violence; MCH, maternal and child health; FHT, Family Health Team; USAID, US Agency for International Development; JICA, Japan International Cooperation Agency; JUST, Jordan University of Science and Technology; IDRC, International Development Research Centre; EHS, Electronic Health Solutions; MODEE, Ministry of Digital Economy and Entrepreneurship; RMS, Royal Medical Services; KHCF, King Hussein Cancer Foundation; RHAC, Royal Health Awareness Society; PHA, Private Hospitals Association; KAFD, King Abdulla II Fund for Development; KAUH, King Abdullah University Hospital; HRP, Humanitarian Response Plan.

#### 3.1.1. Health information systems

The HISs identified in both countries operated at the national level and covered a wide range of services beyond RMNCH. In Palestine, Avicenna is the main national HIS that started in 2010 in public hospitals, aiming to integrate the health data of the patients to coordinate healthcare services at different levels. Avicenna is owned by the Palestinian Ministry of Health (MOH) and has been implemented with funds from several agencies. In Jordan, Hakeem is the main national e-health system that facilitates and implements electronic health records (EHR) in the public health sector. It enables clinicians to electronically access the health records of the patients, both in hospitals and in primary healthcare centers. Hakeem was implemented and funded by Electronic Health Solutions (EHS) in 2009.

In 2009, the UNRWA implemented an electronic health records system (EHS) based on the Family Health Team (FHT) approach. This EHS aimed to improve the quality of services and support the health services of the UNRWA for common illnesses, maternal and child health (MCH), laboratory, and pharmacy. This was implemented in both Palestine (West Bank and Gaza) and Jordan.

Two institutional HISs were also identified in Jordan. These were I-Soft, which is used at King Abdullah the First University Hospital (KAUH), and CertaCure. The Health Technology System at KAUH (I-Soft) is comprehensive, covering clinical and administrative activities. I-Soft is a fully integrated EHR software solution for providers. CertaCure, which is mainly used in the private hospital sector, was developed as a comprehensive, integrated hospital information system to manage clinical and non-clinical information.

#### 3.1.2. Registries

One of the main specific digital RMNCH initiatives in Palestine is the maternal and child health e-registry (MCH e-registry), which started in late 2014, by the Palestinian National Institute of Public Health (PNIPH) in partnership with the Norwegian Institute of Public Health (NIPH). This e-registry aimed to automate data collection of mother, newborn, and child services at the public primary healthcare centers in West Bank and Gaza and to transfer data from the facilities to the national level.

As a support of the national mammogram screening program offered by the MOH for women over 40 years or women at-risk of breast problems, the mammogram e-registry was developed. This e-registry, which started in 2013 by the PNIPH in close cooperation with the MOH, aims to help in monitoring and tracking of the information about all women undergoing mammogram screening. The patient file is saved in a centralized electronic system that notifies patients of their forthcoming appointments via short message service (SMS), increasing women's participation in the program.

As a sub-initiative and part of the Avicenna system, the Near-Miss Registry was introduced in the West Bank in 2015 to monitor and reduce “near-miss” cases among mothers having narrowly survived childbirth to help reduce maternal mortality cases. In addition to the national registries in Palestine, the United Nations International Children’s Emergency Fund (UNICEF) has implemented a global initiative called NutriDash, a global system for collecting and analyzing nutrition information. West Bank and Gaza have been part of this initiative since 2014.

In Jordan, the harmonized reproductive health registry (hRHR) system was developed in 2019 by the Eastern Mediterranean Public Health Network (EMPHNET) in collaboration with Hakeem, as a reproductive health registry for pregnant women and childbirth to improve maternal and child healthcare services in the Al-Mafraq governorate.

#### 3.1.3. Health surveillance systems

In Palestine, there are two main electronic health surveillance systems related to RMNCH. The first is the Maternal Mortality Surveillance, initiated by the Palestinian MOH in the West Bank in 2010 to track the number and causes of maternal deaths. The deaths are registered mainly at the hospitals through Avicenna. Maternal deaths that occur outside the hospitals are captured through the primary healthcare directorate where people submit the death registration form. The second is the National Observatory for Gender-Based Violence (GBVO), announced by the Ministry of Women's Affairs (MWA) in 2018, which is now developed but not yet implemented. This initiative developed in coordination with MOH aims to register, monitor, analyze, and follow up on cases of gender-based violence in all its forms.

We also identified two new health surveillance and registration systems in Jordan, namely, the Jordan’s Maternal Mortality Surveillance and Response System (JMMSRS) and the Jordan Stillbirth and Neonatal Death Surveillance System (JSANDS). The JMMSRS is designed to reduce preventable maternal deaths through a continuous monitoring system which not only registers the number of deaths but also provides information about the underlying causes of deaths and their contributing factors. The JSANDS have been implemented in five major hospitals since August 2019: a university hospital in northern Jordan, a governmental hospital in northern Jordan, a large private hospital in the north of Jordan, a large specialized hospital in Al-Mafraq governorate, and a specialized referral hospital in the southern region of Jordan. This surveillance system was developed as a secure online data entry system to collect, organize, analyze, and disseminate data on stillbirths, neonatal deaths, and related causes. In addition to stillbirths and neonatal deaths, JSANDS also registers all births that occur in each participating hospital to use as a denominator for mortality measures. Definitions of deaths are based on international standards of the WHO and Centers for Disease Control and Prevention (CDC).

#### 3.1.4. Websites

Some initiatives (two in Palestine and one in Jordan) found were in the form of websites which provided information on a wide range of RMNCH services, including appointment booking services and consultations, as well as providing educational and promotional material. In 2016, in Palestine, the Department of Education and Promotion at MOH developed a website and an associated YouTube channel to provide health education materials for mothers and children. In addition, “WebTeb,” a private sector initiative from 2011, provides information on consultations and contact with health professionals. This initiative seeks to make trustworthy medical information available for Arabic readers, connecting citizens, and health information providers worldwide. It has a special focus on MCH services, with a section called “TebBaby” to provide information to mothers throughout the pregnancy and postnatal period. The website provides information to women regardless of their location and authenticity and provides high-quality information. In addition, in 2015, it introduced an associated mobile application, also called “TebBaby,” to help mothers track their pregnancy with relevant information related to pregnancy, delivery, and childcare. The system also generates reminders and information on important tests needed, relating to nutrition, sleeping, and other relevant issues.

In Jordan, we identified a website called “The Breastfeeding Support Association,” a first website in Arabic language, focusing on breastfeeding and related concerns/issues. It disseminates all related information about breastfeeding to mothers, including scientific articles, videos, and pictures related to breastfeeding.

#### 3.1.5. Mobile applications/SMS

We identified three standalone mobile application initiatives and four mobile applications that are associated with other larger initiatives (HISs or health surveillance systems).

In Palestine, there are two mobile applications. The first is “Nabd Al-Hayat” application, launched in 2018 by the Arab American University of Palestine (AAUP) in collaboration with the MOH and World Vision Foundation, to provide interactive information for mothers before, during, and after pregnancy. It provides the mother with access to written and audiovisual educational materials. It includes information about the physical and psychological health of mothers and about the care and nutrition of children during the first year of their life. Messages are provided to users in line with their status and information upon registration. The second initiative in Palestine is the Child Protection Helpline 121, developed by Sawa Civil Society in 2004, as a Toll-free line that receives calls and provides support and counseling to male and female children and youth who are experiencing any form of abuse, violence, or neglect. The helpline represents Palestine in Child Helpline International, a global network that includes helplines from 120 countries.

In Jordan, one standalone mobile application was identified “Children Immunization Application (CImA).” CImA was designed to help Syrian refugees in the Zaatari camp to save their immunization and health records to facilitate health information sharing and to remind them about their scheduled vaccine appointments and alert them on missed appointments.

In addition to the standalone applications mentioned above, we identified four mobile applications/SMS associated with other initiatives. In Palestine, as part of the MCH e-registry, a pilot project is currently being implemented by the PNIPH consisting of a SMS service for registered pregnant/postpartum women for appointment reminders. This pilot project checks if such SMS messages can increase timely attendances and quality of care. Second, the mammogram e-registry has also an associated SMS service that reminds women of their forthcoming appointments. Third is the TebBaby mobile application, mentioned previously, supporting the TebBaby website to provide detailed information to mothers.

The fourth mobile application, which was specifically designed for MCH and part of the UNRWA's EHS, is the e-MCH application. This application is a digitalized version of the Mother and Child Health (MCH) handbook that was distributed at UNRWA healthcare centers since 2009 to all Palestine refugee mothers. This fully customized interactive mobile application has an Arabic interface which makes it user-friendly and enables mothers to enter their personal and their children's information in the UNRWA's e-health system. The application sends notifications to mothers about appointment information and alerts, improving communication between healthcare providers and mothers and their children. The application also enhances health literacy of the mother through its education content about newborn and child care. This application started to be implemented for Palestinian refugees in Jordan since 2017 but has not been used in Palestine yet.

### 3.2. Digital aspects of the initiatives

In terms of platforms or software used in the included initiatives, some of the HISs used specially designed technology platforms that enable the collection, processing, and management of the data. This includes Avicenna HIS in Palestine, which depends on a web-based clinical decision support system for patients’ management called AviTracks-DM. This platform is also used to manage the Maternal Mortality Surveillance system and the Near-Miss Registry, sub-initiatives of Avicenna.

The UNRWA also used an electronic medical records system to collect patients’ data within the e-health system of the general FHT approach. This system is also connected to the e-MCH handbook, which used the free and open-source “Drupal” content management platform, that can universally be used for building versatile and structured content and integrated digital frameworks. Hakeem, the main HIS in Jordan owned by EHS, depends on an open-source medical system called the Veterans Health Information Systems Technology Architecture (VistA), an internationally used EHR, also utilized in the hRHR. I-Soft EHR, an HIS for hospitals, also utilizes an open framework of services and capabilities, known as the DXC platform, a popular tool used by global healthcare providers.

CertaCure, on the other hand, has developed its own unique platform to provide information technology solutions for health services offered by private hospitals in Jordan. This platform provides comprehensive HIS solutions, picture archiving and communication system (PACS), custom software, and web development.

The included registries all use general open-source platforms. Both the MCH and the mammogram e-registry in Palestine depend on the DHIS2 platform. DHIS2 is an open-source, web-based software platform for data collection, management, and analysis. It is one of the world's largest Health Information Management System (HMIS) platforms, used by ministries of health in 73 low-and-middle-income countries, including national-scale deployments in 60 countries (see dhis2.org). In Palestine, based on WHO's guidelines for RMNCH, the DHIS2 Tracker e-Registry collects, analyzes, and tracks case-based data for maternal and child health. In addition to these registries, the GBVO also depends on DHIS2. For the NutriDash registry, the UNICEF adopted the Azure cloud (Microsoft Azure) in 2015 as a replacement of the older system in order to improve their operational and programmatic efficiency. The two surveillance systems (JMMSR and JSANDS) in Jordan have utilized their own special software. Supplementary Material Table S2 shows the main digital aspects of the initiatives which we can report following our initial research.

### 3.3. Governance aspects of the initiatives

All HISs included in this study collect and process the personal data of the patients, which include demographic, medical, and clinical-related data. Both reproductive health-related registries in the two countries, MCH e-registry and hRHR, as well as the mammogram e-registry also collect and process personal data. NutriDash collects aggregated data about the current nutrition programs related to mothers and children. Moreover, all health surveillance systems included also collect personal data of the patients. On the other hand, most of the websites process personal data mainly relating to disease history, symptoms, and some demographic information, in addition to email address, phone number, usage data (IP address of phone or computer used, operation system, and type of device used), and cookie tracker data. One of the websites does not require the collection of personal data nor directly asks a user for personal information to access services and relies on publishing educational information related to breastfeeding topics. However, depending on the configuration of the website and any other analytical tools it might be using, it could be processing personal data, such as the IP address of the phone or computer used, operation system, and type of device used. The mobile applications included in this study also collect personal data of the patients.

The data controller, i.e., the entity which determines the purpose and means of personal data processing, for the data processing activities in all initiatives included is the main implementer, which in most of the cases is the MOH, the responsible UN agency, or the private company that governs the initiative.

It was not easy to collect information about the data-sharing and privacy policies of the initiatives. If they exist at all, most policies are not publicly available. Personal contact with stakeholders has helped in some cases to get some information about the policies followed to regulate data processing activities, including for data-sharing and for ensuring the privacy and security of patients’ data. Some of the included initiatives, especially the websites and the mobile applications, have their privacy policy published online within the description of the initiatives. However, the HISs, the registries, and the health surveillance systems either had no privacy and/or data-sharing policy, or it was not available to the public. This did not make it easy to analyze the content of these policies. Supplementary Material Table S3 shows the main governance aspects of the initiatives identified so far following our preliminary research.

## 4. Discussion

This paper presents the findings of a mapping exercise that aimed to scan the available digital RMNCH initiatives in Palestine and Jordan, understand their characteristics and level of implementation, and explore how they addressed issues of data governance and privacy. The findings of this study are based on web searches and stakeholder contact as per the methodology used. There might be other systems available in Palestine and Jordan that are in-house development, which are not reported in the literature or the web, but are used to collect data on patients such as RMNCH. Such digital solutions are very important to ensure that RMNCH services are available to women who live in low-resource settings where advanced systems may not be feasible. The mapping showed that there is an increasing use of digital technology in the field of RMNCH services in both countries, which has been evolving rapidly in recent years. This increase, however, is not accompanied by clear regulatory policies especially when it comes to privacy and security of personal data and overseeing the data processing activities.

The information about the initiatives was not always easily and/or publicly accessible. Various techniques were used to collect the required information, over two stages: (1) to identify the initiatives and (2) to collect more detailed information about them. Most initiatives were limited to one stakeholder. We could not identify any initiative that was shared by two or more stakeholders. However, such information may emerge in the future as we continue to explore how these initiatives are implemented in practice and the various actors involved in the data processing, even if they were not publicly declared.

This mapping exercise identified 20 digital RMNCH initiatives: 17 fully implemented programs and 3 pilots. These initiatives were mainly developed based on needs and national priorities; however, they were haphazardly designed and did not follow a national plan or framework. Most of these initiatives were not implemented at a national level, reflecting a lack of central planning at the early stages of development. Research shows that digital health solutions should ideally start with investigating core questions of whether pre-identified levels of quality and accessibility are addressed. These include technical functionality and feasibility checks, user satisfaction assessments, and evaluation of the intervention's effectiveness including “value for money” (1, 23).

### 4.1. Purpose, maturity, and evaluation of the digital RMNCH initiatives

While digital RMNCH initiatives are primarily used to support the health of women and children, they are not necessarily effective in improving quality and access to services. A recent systematic review retrieved 245 studies of RMNCH m-health interventions including 51 randomized control trails (RCTs) and showed a positive impact of RMNCH m-health on exclusive breastfeeding and antenatal care. However, almost half (43%) of the RCTs showed the negative or unclear impact of m-health interventions (24). One possible reason could be the lack of rigorous evaluation. This was the case in our data, which showed that even though 11 initiatives have been fully implemented in both countries (Supplementary Material Table S1), they have not been fully evaluated yet (Supplementary Material Table S2). Moreover, these programs are evolving through various stages of maturity life cycle. Nevertheless, legal frameworks that support the implementation of m-health interventions are absent in several countries (1). Also, while electronic health data provide several avenues to evaluate various aspects of health systems, the data are usually poor due to missing vital records and inconsistency in the reported information. Low-quality data hinder the trusted use of these data, particularly in evaluating these initiatives (25). Previous studies showed that RMNCH can be effective in improving the health of women and children only when informed by focused and rigorous monitoring and evaluation of programs and supported by evidence, thus ensuring the effective use of RMNCH m-health interventions (24, 26, 27).

### 4.2. Evidence of increased effectiveness

Digital health solutions have shown that they can improve health outcomes effectively if institutionalized or routinely used as a regular practice in services delivery (28). It has been recommended that robust evidence is needed to identify the value and effectiveness of these programs and to move forward with the scale-up on the national level (1, 24, 28). Thus, in Palestine and Jordan, the actual benefits of such programs are yet to be seen. However, demonstrating the effectiveness of these initiatives is a challenge, probably due to the lack of unified assessment and evaluation criteria as well as lack of collaborative efforts among different parties and stakeholders. The assessment of effectiveness of the initiatives could be done in different ways depending on the type of initiative, for example, this could be through assessing the engagement level on the education and promotion videos or by the number of downloads for mobile applications. Other challenges obstructing the adoption of digital solutions in Palestine and Jordan include limited infrastructure, lack of government commitment, lack of skilled HIS professionals and experts, and lack of funding (16, 25).

While there are a relatively limited number of digital RMNCH initiatives in both countries, a more challenging issue is the fragmentation and incoordination of efforts, to the extent that most of them do not build on the achievements of others. This fragmentation could be a result of lack of communication among these local RMNCH initiatives as well as lack of integration with other available HISs in the two countries. Although the MOH in both countries are responsible for managing health data and information, there is still no national steering committee for the overall responsibility of coordinating with health strategic initiatives at the national level (25). This could also explain the lack of robust evaluation of these initiatives locally.

### 4.3. Priorities identified: addressing accountability, data governance, and human rights

Our initial observations are that at the national level, RMNCH programs should focus on increasing accountability, data governance, and human rights. The responsibility of the data processing activities in all initiatives included in both countries falls on the main implementer, i.e., the data controller, which in most cases is the MOH, UN agency, or the private company that governs the initiative.

Jordan does not yet have a comprehensive data protection law although a draft bill is being discussed in the Parliament, but the current version falls short of internationally recognized principles and standards (29). The situation in Palestine is complex with the West Bank, Gaza Strip, and East Jerusalem subject to different legal regimes. The Palestinian Authority (PA) has not adopted a data protection law, and in despite instances where some parts of Palestine are subject to Israeli laws, they are not protected by Israel's Protection of Privacy Law (30). In addition to this legal void while many if not all of the digital initiatives process the personal data of the patients, it was not easy to find information about the data processing and privacy policies of the included initiatives. From the information collected so far, it appears that the regulation of processing of personal data as per internationally recognized privacy and data protection principles and standards is inexistent in the form of national data protection framework in both countries, and if it exists, it remains weak at the level of regulating each digital initiative.

Various technical features and design choices of these digital initiatives all trigger a variety of questions about the security of the data, the infrastructure, and the measures taken to protect the data and data subjects, i.e., whether the data is encrypted, how permissions are managed, whether the data is protected at rest and in transit, etc. Exploring these technical features will be the focus of future research as part of this research project.

Effective privacy and data protection laws are essential to protect health-related data held in m-health interventions because they identify the rights of users and the responsibility of data controllers and processors to gather, access, and share sensitive information across boundaries (1, 31, 32). A survey conducted in 74 Commission on Information and Accountability (CoIA) countries assessing the privacy safeguards of digital health-related data of women and children found less extensive privacy protection, with only 20% of the these countries having specific legislation on this issue (1). As noted above, Palestine and Jordan are part of those countries without a comprehensive data protection law.

Another important aspect of the legislation to regulate the processing of personal data is related to overseeing and monitoring who can share and access health-related data across the health sector and beyond local geographical boundaries (1) to ensure that the rights of individuals are protected during the data life cycle and prevent function creep among other reasons. Currently, there is a scarcity of legislation that regulates sharing health-related data among national healthcare settings through HIS. Our mapping exercise is congruent with findings from several countries that revealed a lack of nationally adopted standards such as the International Classification of Diseases (ICD) and the Systematized Nomenclature of Medicine (SNOMED) Clinical Terms for the systematic adoption of m-health initiatives and sharing health data (1, 33, 34). Introducing appropriate privacy legislation for health purposes raises benefits and challenges. Of the benefits are the ability to share EHR to enable continuity of care and improved clinical decision-making, and there are also benefits in giving patients control of their own health records, whether these are paper or digital (1, 35). On the other hand, some of the privacy policy concerns for RMNCH include the secondary uses of data which go beyond the original purpose for which the data was collected and which the individual was informed about, who has the permission to access it, and how it is used.

The lack of existing or publicly available privacy policies reflects a general disregard by data controllers for their responsibilities flowing from deploying digital initiatives and processing personal data and a complete lack of understanding and consideration for the effect this may have on the effective protection of people and their data. Their inaction amounts to a failure to identify the risks and the safeguards that must be put in place to ensure those protections. This has implications for the delivery of effective digital RMNCH services.

Furthermore, this information must be assessed in context. These digital initiatives, consisting of public and also public–private partnerships, exist in two countries where no data protection laws are in place and where third parties enjoying privileges and immunities such as the UNRWA are involved. This raises relevant questions about the roles and responsibilities of those processing the personal data and the accountability mechanisms they are subject to.

Further research will be undertaken as part of this project to analyze the existing privacy policies of some of the initiatives included in the study against internationally recognized data protection and human rights standards and principles, and for a few (depending on resources and access), there will be analysis to cross-check the policies with the practice. For those digital initiatives which do not have privacy policies, we will endeavor to identify if any other policies exist to regulate the processing of personal data and how choices are made in terms of privacy and security and guarantees made to users about the protection of their rights in such a regulatory void.

### 4.4. Study strengths and limitations

This study provides compiled information about digital health initiatives related to RMNCH in Palestine and Jordan and analytical research of how these operate in practice and are regulated. We hope it will serve to help policymakers in planning future digital initiatives which are effectively governed to guarantee human rights, gender equality, and effective and equitable access to healthcare and avoid repetition and learn from other experiences. The paper has also identified governance areas for digital health initiatives that need to be discussed and become more visible for the implementers, users, and patients.

The main challenge that this study faced was the availability of information about the digital health initiatives. The researchers have utilized several methodologies and searched several sources to collect as much information as possible. We will continue to endeavor to overcome this challenge by using diverse and innovative research and data collection techniques, but it must be acknowledged that our findings and analysis may be limited to the information we have been able to access within the scope of this study, and further inaccessible information may lead to other findings and conclusions. If further information is brought to our attention and/or becomes accessible over the course of this study, we will ensure to reflect and integrate these new elements to the extent possible given our timeframe and resources.

## 5. Conclusion

Digital health is becoming a part of the health system in both Palestine and Jordan. Digital RMNCH initiatives identified in both countries might be promising to ensure effective and equitable access to RMNCH, but more efforts are needed to understand the implications of such initiatives for data governance and human rights and the need to effectively regulate them. Due to the rapid technical changes in m-health and digital technologies and the resulting unprecedented ways of generating and analyzing health data, the existing policies and regulations, where they are even in place, are insufficient to protect people, their data, and their rights. Further, there is much more needed to be done to enhance inter-sectoral coordination and collaboration to promote m-health interventions to save the lives of women and their children especially in fragile settings. Forming a functional national steering committee which will be in charge of digital solutions coordination is a priority in the two countries. Moreover, data privacy and protection laws and policies should be developed to address all aspects of health data and information including access, storage, retrieval, analysis, and dissemination.

## Data Availability

The original contributions presented in the study are included in the article/Supplementary Material, further inquiries can be directed to the corresponding author.
