# Supplementary material for: Personal data governance and privacy in digital reproductive, maternal, newborn, and child health initiatives in Palestine and Jordan: a mapping exercise
Source: Front Digit Health. 2023 May 25;5:1165692. doi: 10.3389/fdgth.2023.1165692 (PMC10248806; doi:10.3389/fdgth.2023.1165692)
Supplement: Supplementary file 1 [file Table1.docx]

Supplementary Material

Personal Data Governance and Privacy in Digital Reproductive, Maternal, Newborn, and Child Health Initiatives in Palestine and Jordan: A Mapping Exercise

Maysaa Nemer*, Yousef S. Khader, Mohammad S. Alyahya, Alexandrine Pirlot de Corbion, Sundeep Sahay, and Niveen ME Abu-Rmeileh

*** Correspondence:**

Maysaa Nemer

[mnemer@birzeit.edu](mailto:mnemer@birzeit.edu)

# Supplementary Tables

## Supplementary Table 1: Additional Resources on the Initiatives

For more information about the digital RMNCH initiatives in Palestine and Jordan that are included in the paper, the following table includes links to the websites or social media of the initiatives, and links to available research done to describe or evaluate the initiatives.

| Initiative Name | Website / Social media link | Links to research related to the initiative |
| --- | --- | --- |
| Avicenna System | <http://site.moh.ps/> | Building a research registry for studying birth complications and outcomes in six Palestinian governmental hospitals.  <https://bmcpregnancychildbirth.biomedcentral.com/articles/10.1186/s12884-017-1296-6>  Nurses' Attitudes Toward the Use of an Electronic Health Information System in a Developing Country.  <https://journals.sagepub.com/doi/pdf/10.1177/2377960819843711> |
| Family Health Team Approach and the E-health System reform- UNRWA | <https://www.unrwa.org/tags/e-health> |  |
| HAKEEM | <https://ehs.com.jo/hakeem-program>  <https://www.instagram.com/ehsjordan/>  <https://www.linkedin.com/company/electronic-health-solutions>  <https://twitter.com/EHS_jo>  <https://web.facebook.com/ElectronicHealthSolutions?_rdc=1&_rdr> | An Integrated Success Model for an Electronic Health Record: A Case Study of Hakeem Jordan,  <https://www.sciencedirect.com/science/article/pii/S2212567115005262>  The Use of Electronic Medical Records in Jordanian Hospitals  <https://journals.lww.com/cinjournal/Abstract/2017/10000/The_Use_of_Electronic_Medical_Records_in_Jordanian.8.aspx>  Construction of Extended Technology Acceptance Model of Electronic Medical Records in Jordan: The Influence of Doctors’ Self-Efficacy and Perceived Behavioral Control :  <http://www.kmice.cms.net.my/ProcKMICe/KMICe2010/Paper/PG714_719.pdf>  Challenges to healthcare information systems development: The case of Jordan  <https://www.tandfonline.com/doi/abs/10.1080/20479700.2019.1658159>  Barriers and facilitators to using electronic healthcare records in Jordanian hospitals from the nurses’ perspective: A national survey  <https://www.tandfonline.com/doi/abs/10.1080/17538157.2017.1353998>  Evaluating EHR and Health Care in Jordan According to the International Health Metrics Network (HMN) Framework and Standards: A Case Study of Hakeem  <https://ieeexplore.ieee.org/abstract/document/8693495> |
| Electronic Mother and Child Health Handbook (e-MCH) | <https://www.unrwa.org/newsroom/press-releases/jica-and-unrwa-start-digitization-maternal-and-child-health-handbook-jordan>  <https://play.google.com/store/apps/details?id=com.booking.mch&hl=ar&gl=US> | Dissemination and implementation of the e-MCH Handbook, UNRWA's newly released maternal and child health mobile application: a cross-sectional study <https://bmjopen.bmj.com/content/10/3/e034885.long>  UNRWA's electronic MCH Handbook application for Palestine Refugees  <https://www.researchgate.net/publication/327177331_UNRWA's_electronic_MCH_Handbook_application_for_Palestine_Refugees> |
| Health Information System at King Abdullah University Hospital (Isoft) | <https://www.linkedin.com/company/dxctechnology>  <https://web.facebook.com/DXCTechnology?_rdc=1&_rdr>  <https://www.youtube.com/DXCTechnology> | A national framework for e-health data collection in Jordan with current practices  <https://www.inderscienceonline.com/doi/abs/10.1504/IJCAT.2019.097118> |
| CertaCure | <https://www.certacure.com/>  <https://web.facebook.com/Certacure-2156169081294558>  <https://ae.linkedin.com/company/certacure> |  |
| Maternal and Child Health e-Registry (MCH e-Registry) | <https://www.pniph.org/en/health_system/maternal-and-child-health-e-registry> |  |
| Mammogram e-Registry | <https://pniph.org/en/health_system/mammography-e-registry> |  |
| NutriDash (UNICEF) | <https://www.unicef.org/nutrition/index_resources.html> |  |
| Harmonized Reproductive Health Registry (hRHR) | <https://ehs.com.jo/> |  |
| Jordan’s Maternal Mortality Surveillance and Response System (JMMSR) | <https://www.moh.gov.jo/> | Using Data to Combat Maternal Mortality in Jordan  <https://www.abtassociates.com/who-we-are/news/feature-stories/using-data-to-combat-maternal-mortality-in-jordan>  A Model for the Jordan Maternal Mortality Surveillance and Response System (JMMSRS) - Arabic Version  <https://jordankmportal.com/resources/a-model-for-the-jordan-maternal-mortality-surveillance-and-response-system-jmmsrs-arabic-version> |
| Jordan Stillbirths & Neonatal Deaths Surveillance System (JSANDS) | <http://www.jsands.jo/>  <https://web.facebook.com/Jordan-Stillbirths-Neonatal-Deaths-Surveillance-Auditing-System-421520965085888?_rdc=1&_rdr>  <https://twitter.com/jsands_jo>  <https://www.linkedin.com/company/jsands> | The quality of maternal-fetal and newborn care services in Jordan: a qualitative focus group study,  <https://link.springer.com/article/10.1186/s12913-019-4232-9>  JSANDS: A Stillbirth and Neonatal Deaths Surveillance System  <https://ieeexplore.ieee.org/abstract/document/9035335> |
| Electronic page and YouTube channel for educational and promotional material for MCH | <https://www.youtube.com/channel/UCqWFZhHYHrsm_3zuuAX2qzw> |  |
| Webteb Website | <https://www.webteb.com/> |  |
| The Breastfeeding Support Association Website | <https://www.youtube.com/channel/UCVVVo2cGkErYbKVI4F_KA9Q>  <https://www.instagram.com/feedingbreast98/>  <https://web.facebook.com/breastfeedingassociation/?_rdc=1&_rdr> |  |
| Nabd Al- Hayat Application | <https://play.google.com/store/apps/details?id=com.newline.nabd&hl=ar&gl=US> |  |
| Sawa 121"Child protection helpline 121" | <http://sawa.ps/> |  |
| Children Immunization Application (CImA) | <https://play.google.com/store/apps/details?id=com.letsnurture.vaccination&hl=en&gl=US> | Children Immunization App (CImA) Among Syrian Refugees in Zaatari Camp, Jordan: Protocol for a Cluster Randomized Controlled Pilot Trial Intervention Study  <https://www.researchprotocols.org/2019/10/e13557/> |

## Supplementary Table 2:

**Table 2.** Digital aspects of the digital Reproductive, Maternal, Newborn, and Child Health (RMNCH) initiatives in Palestine and Jordan

| No. | Initiative | Software/Platform | Hosting the applications/place of the servers | Managing the servers | Data flow | Level of maturity |
| --- | --- | --- | --- | --- | --- | --- |
|  |  |  |  |  |  |  |
| 1 | Avicenna System | AviTracks-DM, a web-based clinical decision support system for managing patients. | Internal server | IT specialists at MOH | Data entry occurs at hospital and some PHC clinics level. Indicators are generated to be included in the annual report. | Fully developed and implemented in 11 hospitals out of 12 |
| 2 | Family Health Team Approach and the E-health System reform-UNRWA | UNRWA’s e-Health (electronic medical records) system. | Internal server | IT specialists at UNRWA | Data are collected in the United Nations Relief and Works Agency (UNRWA) clinics at district level through the UNRWA’s electronic medical records system. Indicators are generated to be included in the annual report. | Fully developed and implemented |
| 3 | HAKEEM | VistA | Internal server | EHS company | Electronic medical record | Fully developed and being implemented. HAKEEM is implemented in 32 (out of 118) hospitals, 59 comprehensive healthcare centers, and 105 primary healthcare centers. |
| 4 | Electronic Mother and Child Health Handbook (e-MCH) | Drupal – open Source CMS (Drupal.org) (MySQL & PHP).  This was supported by two technology platforms: Jordan’s high smartphone subscription and UNRWA’s e-Health (electronic medical records) system, which can be connected to e-MCH. | Internal server | IT specialists at UNRWA | Data are collected in the United Nations Relief and Works Agency (UNRWA) clinics at district level through the UNRWA’s electronic medical records system. Indicators are generated to be included in the annual report. | Fully developed and implemented |
| 5 | Health Information System at King Abdullah University Hospital (Isoft) | DXC platform | Internal server | IT specialists at KAUH | Electronic medical record | Fully developed and implemented |
| 6 | CertaCure | CertaCure platform | Each hospital has its own server that run its own HIS. | Each hospital mange its own servers | Electronic medical record | Fully developed and being implemented |
| 7 | Maternal and Child Health e-Registry (MCH e-Registry) | Web-based open-source software: District Health Information Software (DHIS2) | Internal server | IT specialist at MOH | Data are collected at PHC clinics level. Indicators generated to be included in the annual report | Fully developed and mostly implemented (In 340 clinics out of 375 clinics) |
| 8 | Mammogram e-Registry | Web-based open-source software: District Health Information Software ( DHIS2) | Internal server | IT specialist at MOH | Data are collected at PHC clinics level. Indicators generated to be included in the annual report | Fully developed and implemented |
| 9 | NutriDash (UNICEF) | A cloud computing service created by "Azure Microsoft " | External server | IT specialist at UNICEF | Data entry is done by the Ministry of Health (MOH), Ministry of Education (MOE), and Non-governmental organizations such as United Nations Relief and Works Agency (UNRWA) on an online and global database to create indicators and annual reports from different countries | Fully developed and implemented |
| 10 | Harmonized Reproductive Health Registry (hRHR) | EHS/ Hakeem platform | External server | EHS company | The hRHR Web-based Application provide a real-time Mother and Child Health (MCH) data for authorized healthcare providers. The application runs in synchronization with EHS/Hakeem core system in which physicians and midwives collect MCH data using digitalized MCH files. Data are stored on EHS/Hakeem servers, whereby access is provided to authorized stakeholders and users. | In progress (The project is a pilot to be implemented in Al-Mafraq governorate). Fully developed and implemented in one healthcare center and in progress to be implemented in others. |
| 11 | Maternal Mortality Surveillance | AviTracks-DM, a web-based clinical decision support system for managing patients. | Internal server | IT specialist at MOH | Maternal death occurring outside hospitals is captured at the PHC directorate where people submit the death form. Hospital deaths are registered through Avicenna system. Indicators are generated to be included in the annual report. | Fully developed and implemented |
| 12 | National Observatory for Gender Based Violence (GBVO) | Web-based open-source software that is District Health Information Software (DHIS2) | Internal server | IT specialist at MWA & MOH | Data are collected from several civil institutions that receive cases of Gender Based Violence (GBV). Indicators generated to be included in the annual report | Fully developed but not implemented |
| 13 | Jordan’s Maternal Mortality Surveillance and Response System (JMMSR) | JMMSR software using .Net Microsoft technology | Internal servers | IT specialist at MOH | Notification of all deaths among women of reproductive age is carried out within 24 hours by trained focal points at the reporting sites. Once deaths among women of reproductive age are notified, the assigned Directorate Advisory Group (DAG) members identify which deaths were maternal. Once a case was identified as a maternal death case the MDR is conducted to collect data on demographic characteristics, information on the pregnancy, information on delivery, antenatal care, death, time of death, causes of deaths, contributing factors etc.  The DAG worksheet must include a case summary that describes the main highlights of the circumstances surrounding each death and the final DAG decision. | Fully developed and implemented |
| 14 | Jordan Stillbirths & Neonatal Deaths Surveillance System (JSANDS) | JSANDS software | Internal servers | JUST | Stillbirths and neonatal deaths are identified by health care providers mainly obstetricians, pediatricians, and nurses who confirm the death and cause of death. Then the relevant data are entered into JSANDS that will be automatically transferred to MOH where reports are produced and disseminated to different audiences. | In progress. Fully developed and being implemented in five hospitals and in progress to be scaled up. |
| 15 | Electronic page and YouTube channel for educational and promotional material for MCH |  | Internal & External server | IT specialist at MOH | The educational materials are prepared by the department of education and promotion in the Ministry of Health (MOH). Then individuals (targets) can see it via Electronic page on the MOH website or YouTube channel specific for MOH. | Fully developed and implemented |
| 16 | Webteb Website |  | External server | IT specialist at Webteb | The medical and health information exists on the website and users can open the link, search and read. | Fully developed and implemented |
| 17 | The Breastfeeding Support Association Website | Website | Internal server | The breastfeeding support association | The website does not collect personal or clinical data; it disseminates educational and promotional information. | Fully developed and implemented |
| 18 | Nabd Al- Hayat Application |  | Internal server at MOH & AAUP | IT specialist at AAUP & MOH | Data entry is at individual level (pregnant women) to receive health information according to their situation. | Fully developed but not implemented (piloting in two cities) |
| 19 | Sawa 121"Child protection helpline 121" | Independently developed database by Sawa | Internal server | IT specialist at SAWA | Data entry is on separate database and only occasional reports sent to ministries or other institutes. | Fully developed and implemented |
| 20 | Children Immunization Application (CImA) | Technology Stack was designed for Android. The design tools/software included Android Studio 3.4. The app language was Kotlin. The Operating System (OS) included Android 4.0 and above. The Database (local) was SQLite.  Regarding web-interface, the web development language/framework was Laravel 5.5, and MySQL was used for web development (website/backend). | iCloud | The project manger |  | Fully developed and implemented as a trial that carried out at the Zaatari refugee camp in Jordan. |
| Abbreviations | | DHIS2: District Health Information Software; MCH: Maternal and Child health; AAUP: Arab American University Palestine; UNRWA: The United Nations Relief and Works Agency for Palestine Refugees; OCHA: United Nations Office for the Coordination of Humanitarian Affairs; EHS: Electronic Health Solutions; JSANDS: Jordan Stillbirths and Neonatal Deaths Surveillance System; DAG: Directorate Advisory Group; MDR: Maternal Death Review | | | | |

## Supplementary Table 3:

**Table 3.** Governance aspects of the digital Reproductive, Maternal, Newborn, and Child Health (RMNCH) initiatives in Palestine and Jordan

| No. | Initiative | Type of  collected data | Data Controller* | Data Sharing Policy | Privacy Policy |
| --- | --- | --- | --- | --- | --- |
|  |  |  |  |  |  |
| 1 | Avicenna System | Personal demographic data  (age, gender, etc.), and other medical, clinical or health related data (obstetric, birth, death, etc.) | Ministry of Health (MOH) | Yes, but not available online. It is available as a hard copy | Yes, but not available online. It is available as a hard copy |
| 2 | Family Health Team Approach and the E-health System reform-UNRWA | Personal demographic data (age, gender, etc.), and other medical, clinical or health related data (obstetric, birth, antenatal and postnatal, etc.) | United Nations Relief and Works Agency (UNRWA) | Yes, but not available online. | No |
| 3 | HAKEEM | Personal demographic data, admission, discharge, and transfer (ADT), diagnosis, allergies, immunization, orders, laboratory tests, surgical history, diet, anesthesia, medication administration, outpatient clinics appointments, radiology, vital signs, and billing system (piloting currently). | EHS company | No | No |
| 4 | Electronic Mother and Child Health Handbook (e-MCH) | Personal data including country, health center, health team, refugee registration information system (RRIS) number for mother. | UNRWA | Yes, but not available online. | No |
| 5 | Health Information System at King Abdullah University Hospital (Isoft) | Personal demographic data, patient billing data, insurance, and clinical data including patient’s chief complaint, medical history, initial diagnosis, lab results, radiological (images and report), medication, consultations, vital signs, final diagnosis, allergies, diagnostic and treatment procedures, treatment plan and follow up plan. | King Abdullah University Hospital (KAUH) | Yes, the hospital has data sharing policy, which includes: Authorized health care providers. | Yes; KAUH hospital maintains the privacy and confidentiality of all data and information and is particularly careful about preserving the confidentiality of sensitive data and information. The balance between data sharing and data confidentiality is addressed. The hospital determines the level of privacy and confidentiality maintained for different categories of information (for example, patient information, research data, and quality data). |
| 6 | CertaCure | Patient’s demographic, medical and administrative (i.e. billing) information, and insurance data and information. Based on the clinical examination the physician entered specific clinical data and orders. Then, requested test results entered by each department or unit. Based on the final diagnosis, the physician prescribe medication for patients electronically. Also, clinical procedures, allergies, treatment plan, and follow up plan entered on the system. | Each hospital that implements the system owns its data. | Yes | Each hospital has its own privacy policy. |
| 7 | Maternal and Child Health e-Registry (MCH e-Registry) | Personal demographic data (age, gender, etc.) and other medical, clinical or health related data (obstetric, birth, antenatal, postnatal, neonatal, lab tests, vital signs, etc.) | MOH | No | No |
| 8 | Mammogram e-Registry | Personal demographic data (age, gender, etc.) and other medical, clinical or health related data (screening for breast cancer, digital breast images results, etc.) | MOH | No | No |
| 9 | NutriDash (UNICEF) | Aggregated data about the current nutrition programs and services such as maternal and child malnutrition, adolescent nutrition, child health problems (stunting, wasting…etc.) | United Nations Children's Fund (UNICEF) | Yes, but not available online | No |
| 10 | Harmonized Reproductive Health Registry (hRHR) | The collected data include antenatal care, postnatal care, family planning, child growth and developments, and child vaccinations. The application runs in synchronization with EHS/Hakeem core system in which physicians and midwives collect MCH data using digitalized MCH files. | MOH | No | No |
| 11 | Maternal Mortality Surveillance | Personal demographic data (age, gender, etc.) and other medical, clinical or health related data (death, cause of death, etc.) | MOH | No | No |
| 12 | National Observatory for Gender Based Violence (GBVO) | Personal demographic data (age, gender, etc.) and for other medical or clinical or health related data (violence against women in all its forms). It includes a high sensitive data. | Ministry of Women's Affairs (MWA) | Yes, but not available online. There is data sharing agreement between Palestinian National Institute of Public Health (PNIPH) and Ministry of Women’s Affairs (MWA). | No |
| 13 | Jordan’s Maternal Mortality Surveillance and Response System (JMMSR) | Personal data, delivery data (date, time, and place of delivery), antenatal care, time of death, cause of death, contributing factors of death. | MOH | Yes. MOH owns the data. According to the policy, MOH share the maternal heath indicators, measures of maternal mortality, and causes of deaths. | Yes, there are JMMRS policies and procedures which contain statements about data privacy and security and data confidentiality. |
| 14 | Jordan Stillbirths & Neonatal Deaths Surveillance System (JSANDS) | JSANDS data include demographic, clinical, and obstetric characteristics, birth characteristics (Birthweight and GA), death, time of death, causes of deaths. Etc. | MOH | Yes, MOH owns the data. According to the policy, MOH share the maternal and child heath indicators, measures of mortality, and causes of deaths. The raw data could be shared according the data sharing policy. | Yes, there are data privacy, confidentiality, research and data sharing policies. |
| 15 | Electronic page and YouTube channel for educational and promotional material for MCH | Educational and promotional information, videos and pictures about maternal and child health for pregnant women and for health care providers. | MOH | No | No |
| 16 | Webteb Website | Personal data for users (email name, phone number), usage data (IP address for your phone, type of your phone, operating system) and cookie tracker data for your browsing or security or advertisements. | Web Medicine limited Company | No | Yes, privacy policy and the terms of use exist on a website. It explain the type and usage of personal data collected, the human rights perspectives regarding it. |
| 17 | The Breastfeeding Support Association Website | Educational and promotional information including articles, videos and pictures related to breastfeeding topics. | No collected data | No, the content of the website is public and can be browsed anonymously. | Yes  -Association of breastfeeding mothers’ comments, compliments, and complaints procedure.  -Equal opportunities, diversity and discrimination policy. |
| 18 | Nabd Al- Hayat Application | Educational information, some personal and demographic data (age, period, stage of pregnancy but they are not obligatory questions) | MOH, Arab American University Palestine (AAUP) | Yes, but not available online. There is a data sharing agreement between MOH and AAUP | No |
| 19 | Sawa 121"Child protection helpline 121" | Personal data such as demographic data (age, gender, etc.) and other sensitive data about violence, abuse, or other forms of mistreatment and distress. Aggregated data are collected too. | Sawa | Yes, but not available online. | Yes, it exists in the annual report of Sawa and taking about the server access and the communication process with cases. |
| 20 | Children Immunization Application (CImA) | Socio-demographics and perception of smartphones and vaccines. | The collected data belongs to the project (pilot). | No | No |
| Abbreviations | | MOH: Ministry of health; PNIPH: Palestinian national institute of public health; MWA: Ministry of Women's Affairs; UNRWA: United Nations Relief and Works Agency; UNICEF: United Nations Children's Fund; UN: United Nations; MCH: Maternal and Child health; AAUP: Arab American University Palestine; OCHA: United Nations Office for the Coordination of Humanitarian Affairs, RRIS: Refugee Registration Information System ; CMS: Content Management System: JSANDS: Jordan Stillbirths and Neonatal Deaths Surveillance System; ADT: Admission, Discharge, and Transfer; EHS: Electronic Health Solutions; KAUH: King Abdullah University Hospital; DAG: Directorate Advisory Group; MDR: Maternal Death Review | | | |
| * Data controller: the entity which determines the purpose and means of personal data processing | | | | | |
